# Role of mordenite zeolite in improving nutrient and water use efficiency in Alfisols

**DOI:** 10.3389/fpls.2024.1404077

**Published:** 2025-01-30

**Authors:** Girijaveni V, Sammi Reddy K, Srinivasarao Ch, Raju B M K, Divya Balakrishnan, Sumanta Kundu, Pushpanjali, Jagriti Rohit, Singh V K

**Affiliations:** ^1^ Indian Council of Agricultural Research (ICAR)-Central Research Institute for Dryland Agriculture, Hyderabad, Telangana, India; ^2^ ICAR-National Institute of Abiotic Stress Management, Pune, Maharashtra, India; ^3^ ICAR-National Academy of Agricultural Research Management, Hyderabad, Telangana, India; ^4^ ICAR-Indian Institute of Rice Research, Hyderabad, Telangana, India

**Keywords:** Alfisols, crop production, food security, mordenite zeolite, NUE, WUE

## Abstract

Poor nutrient use efficiency (NUE) and water use efficiency (WUE) is a predominantly faced problem in semi-arid regions that limit the crop production. This problem can be addressed with the application of zeolite that is a naturally available mineral with very high cation exchange and water holding capacity, which aids in improving NUE and WUE. Moreover, zeolites are safe for the environment and living organisms, and their use in agriculture results in improving physical and chemical properties of soil. Yet, its study is very limited in semi-arid regions of India. Thus, a study was conducted with locally available zeolite at CRIDA, Hyderabad. Zeolite was further characterized using X-ray diffraction (XRD) and SEM, as the type of zeolite collected is unknown from local market. The results of XRD and SEM revealed that the zeolite collected was mordenite zeolite. Our study includes laboratory and pot experiment where laboratory includes sorption and leaching column study to evaluate the zeolite capacity to hold and release the nutrients especially NH_4_^+^, P, and K. In this study, the adsorption behaviour of the natural mordenite was examined, and it was found that the maximum adsorption capacity for NH_4_^+^, P, and K were estimated as 10.6, 1.08, and 2.15 mg g^−1^, respectively, suggesting the zeolite has good affinity for N. Furthermore, the column study revealed that there was 15.4% reduction in NH_4_^+^–N loss with 10 tonnes zeolite ha^−1^ + N @ 100 kg ha^−1^ as compared to N alone, while the reduction was 39.6% with 10 tonnes zeolite ha^−1^ + N @ 500 kg ha^−1^ compared to N alone, suggesting that the zeolite could control the release of N as compared to the sole application of N, which was supplied through urea. In addition, pot experiment was carried out with three levels of fertiliser rates, four levels of zeolite, and two levels of moisture in randomised complete block design with three replications to evaluate the changes in soil available nutrients and their uptake in tomato. Results revealed that there was a significant positive impact on yield, water use efficiency, nutrient (N, P, and K) uptake, and soil available nutrients. Highest soil available N, P, and K, crop uptake, and yield were observed due to zeolite application @ 200 kg ha^−1^ along with 100% recommended dose of fertilization in Alfisols. Thus, zeolite application along with chemical fertilisers can improve the nutrient availability by reducing the leaching losses and improving nutrient use efficiency.

## 1 Introduction

Sustainable management of soil and water in the climate change scenario is the key to feed the ever growing human population. Obviously, with the increase in world population at a rapid rate, there would be huge demand for food, shelter, etc. With the limited resources of land and water, which are degrading at faster rate, it is necessary to ensure food security with improved soil health, nutrient use efficiency, and water use efficiency. Moreover, the major soils in semiarid tropics belong to Alfisols. In FAO/UNESCO classification system, Alfisols are comparable to Luvisols. They usually have a base-rich argillic B horizon representing the largest single soil group of the semi-arid tropics. In India, Alfisol soils cover 79.7 m ha, characterized by red, reddish brown to yellowish brown colours. Most of the Alfisol soils are at the verge of degradation, having low cropping intensity, relatively low organic matter status, poor soil physical health, low fertility, etc. Due to poor nutrient use efficiency, nutrient losses due to leaching, volatilization, and denitrification are high in these soils. In India, due to substantial inter-seasonal variations and monsoon disturbances, the crops grown in these soils fail, as these have low water holding capacity. Hence, various measures need to be taken by leveraging the technology towards enhanced nutrient and water use efficiency especially in semi-arid tropics. One such strategy is the use of zeolite. Zeolite is a natural amendment that can be used along with fertilisers to improve their use efficiency and also water use efficiency in soils.

Zeolites are naturally occurring crystalline-hydrated aluminosilicates of framework structure containing pores occupied by water and alkali and alkaline earth cations (Ramesh and Reddy, 2011; Li et al., 2023). Zeolites are well-known ion exchangers in the world. Among 40 different types of natural zeolites, most commonly used in crop production includes clinoptilotile, erionite, and mordenite (Mpanga et al., 2020). Zeolites are known to ensure NH_4_^+^ retention in soils, preventing leaching of NH_4_^+^ and nitrate (NO_3_^−^) and other losses. The mechanism of adsorption of K^+^ in zeolite similar to NH_4_^+^ and to smaller extent can adsorb phosphate (PO_4_^3−^) being anion. The ability to remove nutrients and their release pattern can be effectively studied through isotherm and leaching column studies. These studies give a clear picture of nutrient supply, as zeolite plays a key role in nutrient cycling through sorption process and leaching when applied to soil. Sorption studies are evaluated using empirical models such as Langmuir, Freundlich, and Temkin isotherms. Langmuir isotherm indicates the amount of nutrients held in the form of a single layer on the surface. Mostly, Langmuir isotherm is suitable to study the single layer surface adsorption reaction for adsorption sites (Kalam et al., 2021). Freundlich isotherm demonstrates the mechanism of adsorbed nutrients and adsorbent surface at different sites or heterogeneous adsorbent surface (Palanivell et al., 2019; Akrawi et al., 2021), while the binding energy involved in adsorbing nutrient is measured by Temkin isotherm (Murphy et al., 2023). Temkin isotherm assumes that heat generation during the process of nutrient adsorption decreases linearly as the adsorbent coverage increases (Ohale et al., 2020). In a study where NH_4_^+^, Na^+^, and K^+^ ions were provided in the solution, it was found that zeolite could remove more NH_4_^+^ and K^+^ ions in comparison with Na^+^, which remained in the solution. In the same study, the efficiency of zeolite and vermiculite was evaluated in removing NH_4_^+^ and found that it was high for zeolite (85%), while it was almost 70% for vermiculite (Shinzato et al., 2020). Several studies found reduced nitrogen leaching due to soil application of zeolites in combination with chemical fertiliser volatilisation (Aghaalikhani et al., 2012; Vilcek et al., 2013; Omar et al., 2015; Nakhli et al., 2017; Cataldo et al., 2021; Mondal et al., 2021).

Research revealed that zeolite can be best soil amendment for soil types with coarse texture, deficit moisture levels, and low fertility levels (Ghorbani et al., 2022). Additionally, its application helped in balancing pH, improving chemical properties, restoring soil microbial activity, increasing moisture retention, and reducing compaction. Zeolites have high retention ability of ammonium and potassium and help to retain nutrients in the root zone. Recent studies show that use of zeolites is beneficial, as it helps in improving the physical and chemical properties of soils. It is mainly due to its higher cation exchange capacity (CEC), higher specific surface area, internal void structure, higher moisture holding capacity, etc. Thus, these properties of zeolites can help in enhancing the water and nutrient use efficiency. In addition, it reduces the risk of environmental pollution occurring due to reduction in nitrate leaching, emissions of nitrous oxides, and NH_3_ (Louhar, 2020). Zeolite application improved nitrogen, phosphorus, and potassium fertilisers use efficiency in Typic Paleudults (Palanivell et al., 2021). Zeolite application maintained higher mineral and mineralisable N in different types of soils (Saha et al., 2018; Mondal et al., 2021; Hazrati et al., 2022). Moreover, the porous nature of zeolite aids in soil aeration, and moisture maintenance in turn improves the crop performance and production.

Despite the great interest in zeolites as agricultural amendment, few data exist with mordenite, in terms of their use in agriculture. Mordenite is an orthorhombic zeolite of high silica content and is a common alteration product of pyroclastic sediment, sedimentary rock, and lava flows of worldwide distribution (Narayanan et al., 2021). The ideal composition of mordenite is [(Na_2_K_2_Ca)_4_Al_8_Si_40_O_96_] 28H_2_O. Its characteristics include high heat stability with specific gravity of 2.12–2.15 g cm^−3^ and bulk density of 1.7 g cm^−3^ with porosity of 28% (Cataldo et al., 2021). Due to soil application of mordenite zeolite, the soil water infiltration increased by 7%–30% on a gentle slope land, while it was increased by 50% on steep slope land (Xiubin and Zhanbin, 2001). Natural and synthetic zeolitic materials of clinoptilolite and mordenite were used as soil amendment and found that amended soils exhibited higher CEC values compared with unamended soil (Giannatou et al., 2018). Mixtures of zeolite tuff (chabazite- and mordenite-rich tuff) were tested as slow release fertiliser in loamy soil. Even in abandoned quarries in semiarid Mediterranean areas where soil fertility and water availability are highly limited, native plant species (*Olea europaea* var. *Sylvestris*, *Pistacia lentiscus*, *Rosmarinus officinalis*, and *Quercus coccifera*) could sustain through the addition of organic (compost derived from horticultural crop residues and poultry manure) and inorganic (three types of zeolites: mordenite, clinoptilolite, and ZeoPro) amendments. Thus, zeolite could play a key role in soil restoration (Ortega et al., 2020). Zeolite addition also improves microbial activity where application of chabazite zeolite showed a positive effect on the scoring of GLU activity, which an indicator of microbial activity in the coarse-textured soil of Perennial-Olive system (Chae et al., 2017; Levakov et al., 2021). In Mediterranean soils, co-addition of 5% compost + 2% zeolite improved the NPK availability over no application of zeolite. The addition of stilbite-zeolite @ 1.25%–10% w/w in a contaminated soil could improve the chemical properties of soil and was effective in remediating the soil; also rye grass studied showed a gradual increase in yield with increase in addition, suggesting that natural zeolite showed a positive impact on crop yield by improving chemical properties and reducing environmental impact of contaminated soil (Amirahmadi et al., 2022). Long-term study with Chabazite-Zeolite @ 5 kg m^−2^ as amendment improved soil quality in arable and perennial cropping systems with significantly higher scores (lower bulk density and NH_3_–N emissions) (Ferretti et al., 2024). Thus, the objective of the study was (1) to evaluate the modernite zeolite for ammonium, phosphorus, and potassium removal through sorption and release through leaching column study and (2) to study its application along with chemical fertilisers on crop uptake and soil nutrient dynamics in Alfisols.

## 2 Materials and methods

**Figure 1:**
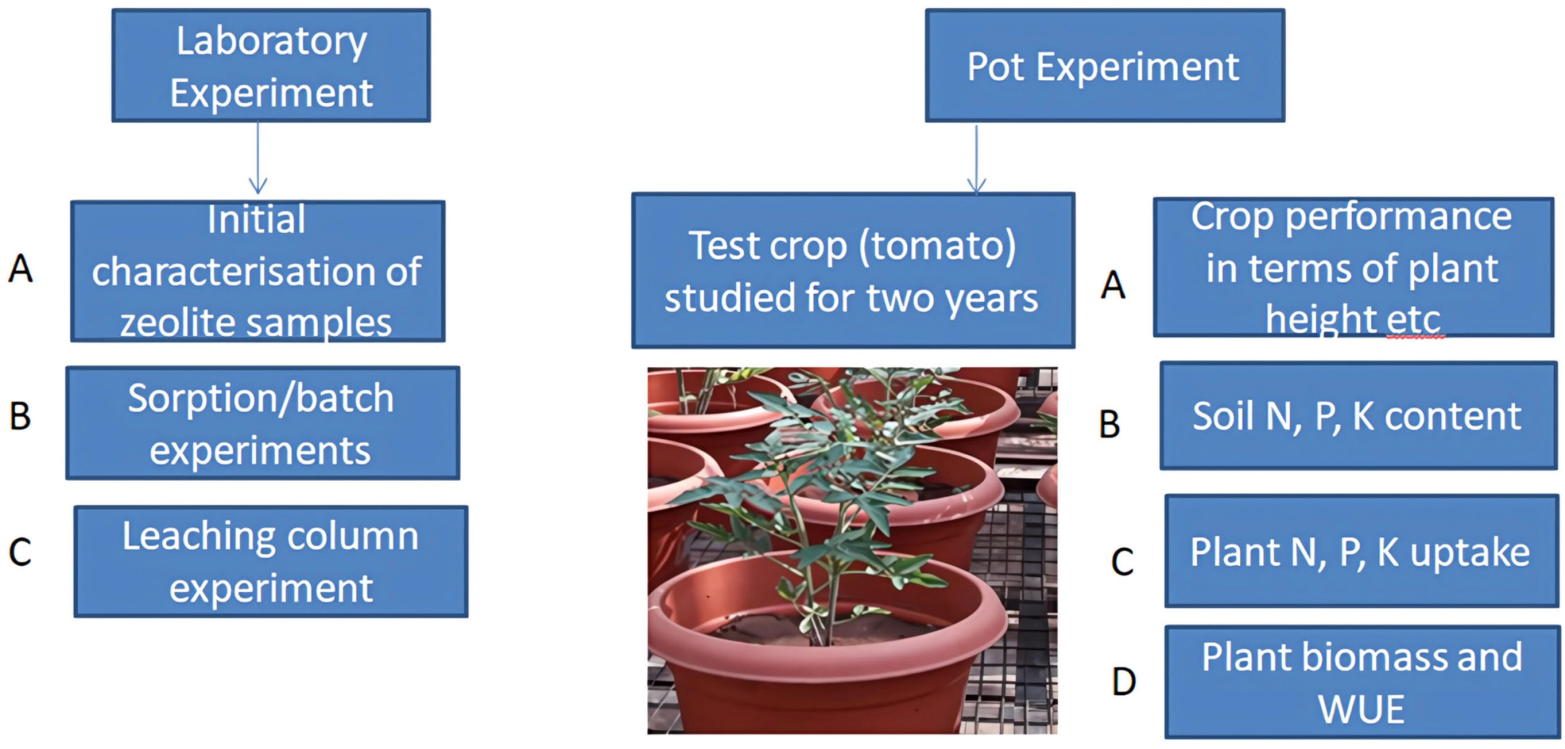
Flow chart showing the research work at a glance.

Natural zeolite available in the local market was purchased, and the study was carried as given below in **Figure 1**.

### 2.1 Laboratory experiment

#### 2.1.1 Characterization

The crystalline phases were identified by X-ray diffraction (XRD) using a D8 Advance X-ray diffractometer. X-ray diffraction analysis (XRD) of the materials was performed using a D8 Advance diffractometer (Bruker AXS) operating at a tube voltage of 40 kV and a tube current of 40 mA. The X-ray beam was filtered with a Ni 0.02-mm filter to select a CuKα wavelength. The specimens were scanned over a 2θ range from 3° to 70° at a scanning speed of 5° min^−1^ using a coupled two theta/theta scan type (Harrington and Santiso, 2021). The identification of zeolite sample was confirmed by matching the powder XRD patterns of the samples with the diffractograms of single-phase patterns from Powder Data File (PDF), compiled by the International Centre for Diffraction Data (ICDD).

The scanning electron microscopy (SEM) images of the zeolite sample were studied where the samples were fixed in 2.5% gluteraldehyde in 0.1 M phosphate buffer (pH 7.2) for 24 h at 4°C and post-fixed 2% in aqueous osmium tetroxide for 4 h (Sandoval-Molina et al., 2017). The samples were dehydrated in series of graded alcohols and dried to critical point drying with CPD (EMS-850) unit by using liquid carbon dioxide. The processed samples were mounted over the stubs with a double-sided carbon conductivity tape, and a thin layer of gold coat over the samples were done by using an automated sputter coater (Model JEOL JFC-1600, Peabody, Massachusetts, USA) for 3 min and scanned under a scanning electron microscope (SEM, Model JOEL-JSM 5600) at required magnifications as per the standard procedures at RUSKA Lab’s College of Veterinary Science, PVNRTVU, Rajendranagar, Hyderabad, India.

#### 2.1.2 Adsorption experiments

Batch experiments were conducted to evaluate the adsorption capacity of zeolite for ammonical-N, phosphorus, and potassium. Batch experiments were conducted in an array of conical flasks, where 100 mL of graded concentrations of ammonium solutions were added to 250-mL Erlenmeyer flasks with 1 g zeolite, sealed and shaken at a speed of 180 rpm for 2 h in a mechanical shaker at 25°C. The influence factors of ammonium and potassium adsorption were studied with the initial ammonium concentration (25–400 mg L^−1^) and initial potassium concentration (25–300 mg L^−1^). An ammonium chloride (NH_4_Cl) salt was used to make the NH_4_^+^ solutions, and potassium chloride (KCl) used to make the K^+^ solutions. The equilibrium ammonium concentration was measured using Nessler’s reagent and phosphorus through blue colour method in spectrophotometry, measured by a UV-VIS spectrophotometer (UV-1800, Shimadzu, Kyoto, Japan) and three replicates were performed in parallel for each set of experiments, while the equilibrium potassium concentration was measured using flame photometer. Differences in the concentration between the initial and residual solution were used to calculate the equilibrated adsorption capacity. The data were fit in the Langmuir and Freundlich isotherm equations as given below:


$${\text{Freundlich equation: it is represented by q}}_{\text{e}}={\text{K}}_{\text{F}}{\text{C}}_{e}^{1/\text{n}}$$

where qe is the adsorbed amount at equilibrium (mg g^−1^) and C_e_ represents the equilibrium concentration (mg L^−1^). K_F_ and n are equilibrium constants indicative of adsorption capacity and adsorption intensity, respectively. If n is greater than 2 and less than 10 (2 < n < 10), the adsorption process is favourable.


$$\text{Langmuir equation}:{\text{q}}_{e=}{\text{q}}_{\max}{\left({\text{K}}_{\text{S}}{\text{C}}_{\text{e}}\right)}^{\text{n}}/1+{\left({\text{K}}_{\text{S}}{\text{C}}_{\text{e}}\right)}^{\text{n}}$$

where qe and Ce are the adsorption capacity at equilibrium and the equilibrium concentration respectively, q_max_ represents the maximum adsorption capacity (mg g^−1^), K_s_ is the adsorption equilibrium constant, and n is the dissociation parameter.

The removal efficiency (R %) and the adsorption capacities of adsorbents at time (qt, mg/g) and at equilibrium (qe, mg/g) were calculated using the following equations:


$$\text{Removal efficiency}\left(\%\right)=\left({\text{C}}_{o}-{\text{C}}_{e}\right)/{\text{C}}_{o}\times 100$$

$${\text{Q}}_{e}\left({\text{mg g}}^{-1}\right)=\left({\text{C}}_{o}-{\text{C}}_{e}\right)/\text{m }\times \text{V}$$

where C_0_ and C_e_ are the initial and equilibrium concentration of the cation (mg L^−1^), V is the volume (L) of solution, and m is the mineral mass (g).

#### 2.1.3 Column study

A soil leaching experiment was conducted to evaluate the inorganic nitrogen leaching for 45 days in the presence of mordenite zeolite in Alfisol of sandy loam texture. The surface soil (0–15 cm) was collected from Hayathnagar Research Farm, CRIDA for this study. Column study with glass columns of 5 cm internal diameter and 50 cm height was used to investigate the release pattern at field scale situation. Columns were organized with three replications. The treatments comprised of two levels of N (100 kg ha^−1^ and 500 kg ha^−1^) and four levels of zeolite (0.2 tonnes ha^−1^, 0.5 tonnes ha^−1^, 5 tonnes ha^−1^, and 10 tonnes ha^−1^). All treatment combinations were thoroughly mixed and scaled down to the amount of 900 g soil. The pore volume for these columns was found to be 981.25 cm^3^. The filled columns were saturated with distilled water and then irrigated homogeneously with known volume of distilled water, and the leachate was collected with an interval of 3 days and analysed for ammonical-N content using Nessler reagent for colour development (Krug et al., 1979).

### 2.2 Pot experiment

A net house experiment was conducted for 2 years (2017 and 2018) to assess the effect of zeolite amendment along with chemical fertilisers on tomato plant growth. This experiment was carried in the net house of Central Research Institute for Dryland Agriculture (CRIDA), Hyderabad. For this experiment, surface soils (0–15 cm) were collected from Hayathnagar Research farm of CRIDA, Hyderabad. The collected soil samples were air dried, processed, and sieved for taking up the pot experiment. The tomato (PKM-1 variety) was used as test crop.

The experimental setup is presented in **Table 1** where there are 12 treatments with a total of 72 pots taken up. The experimental design followed was factorial completely randomised design (CRD) with three replications. The fertiliser and zeolite doses added were scaled down to 10 kg soil taken in the pots. The experiment was laid under two levels of moisture conditions—50% and 100% FC. To maintain the moisture levels, the calculated amount of water was used to irrigate the pots to maintain the field capacity. Fertiliser materials used were urea, diammonium phosphate (DAP), and muriate of potash (MOP). Irrigation and other agronomic practices were carried out as per requirement. Crop yield parameters such as plant height, number of leaves, and biomass yield were recorded, and nutrient status was studied in post-harvest soil samples after the harvest of tomato during both the study years.

**Table 1:** Experimental set up to study the effect of zeolite with chemical fertilisers.

Sl. No.	Factors	Details
1	Factor 1	50% FC; 100% FC
2	Factor 2	Control; 50% RDF; 100% RDF
3	Factor 3	Z0; Z50; Z100; Z200

FC denotes field capacity (%), F denotes recommended dose of fertiliser (kg/ha), and Z denotes zeolite (kg/ha); RDF, recommended dose of fertiliser application; 50% RDF = 90: 50: 25 kg NPK/ha; 100% RDF = 180: 100: 50 kg NPK/ha; Z0 = control; Z50 = 50 kg/ha; Z100 = 100 kg/ha; Z200 = 200 kg/ha.

#### 2.2.1 Soil analysis

The initial soil samples were analysed for mechanical composition, pH, electric conductivity (EC), organic carbon, and available nutrients (N, P, and K) following standard procedures. The physicochemical properties of the initial soil under study are presented in **Table 2**. Soil water retention at permanent wilting point (PWP) and field capacity (FC) were measured in pressure plate apparatus at −1.5 MPa and −0.033 MPa (Cassel and Nielsen, 1986). The difference between PWP and FC was calculated as available water. A representative portion of each soil sample was air dried, powdered, and passed through a 0.2-mm sieve for the determination of organic carbon (OC) by Walkley and Black’s method (Jackson, 2005). Available soil nitrogen was determined by alkaline–KMnO_4_ method given by Subbaiah and Asija (1956), which primarily measures easily oxidizable N using Kjeltec Auto 1030 Analyser made by Tecator in Sweden. Available P (Olsen P) was determined by sodium bicarbonate (NaHCO_3_) extraction and subsequent colorimetric analysis (Olsen et al., 1954). Exchangeable K was measured using the method suggested by Hanway and Heidal, 1952.

**Table 2:** Physicochemical properties and fertility status of the experimental soil before taking up the study.

Soil characteristics	Description
Soil order	Alfisols
Moisture content at field capacity (% w/w)	8.42
Texture	
pH (soil: water=1:2)	6.2
EC (dS m^−1^)	0.091
Organic carbon (g kg^−1^)	3.9
KMnO_4_-N (kg ha^−1^)	112.6
Olsen-P (kg ha^−1^)	15.32
NH_4_OAc-K (kg ha^−1^)	133.7

#### 2.2.2 Plant analysis

Plant samples collected were dried in oven at 65°CC and powdered by using a plant sample grinder. The powdered samples were packed in polyethylene zip covers for further analysis. These samples were used for the estimation of nitrogen, phosphorus, and potassium according to the procedure.

##### 2.2.2.1 Total N (%)

Nitrogen content in the plant sample was determined by micro-Kjeldhal distillation method using Kelplus equipment (Jackson, 1973). To a 0.1 g of powdered plant sample in a test tube, 3 ml of concentrated sulphuric acid was added and predigested for 24 h. The black charred contents in the test tube turned into clear supernatant solution by the addition of 1 ml of 30% hydrogen peroxide against flame. The contents were transferred into a Kjeldahl tube for distillation. A 250-ml conical flask containing 25 ml of 4% boric acid with mixed indicator was placed at the end of delivery tube; 15 ml of 40% sodium hydroxide was run into the Kjeldahl tube automatically after placing the Kjeldahl tube in position. After completion of distillation, the conical flask with distillate is titrated against 0.01 N sulphuric acid until the bluish green colour turned into pink colour. A blank was run simultaneously.

##### 2.2.2.2 Digestion of plant samples

One gram of oven-dried and processed plant samples was digested with a 9:3:1 mixture of nitric acid, perchloric acid, and sulphuric acid on a hot plate. The clear digested residue was cooled, diluted to 100 mL with double distilled water, and filtered to remove insoluble silica.

##### 2.2.2.3 Phosphorus content (%)

In the digested extract, phosphorus content was determined by Vanado–molybdo phosphoric yellow colour method as described by Piper (1966) using a UV-VIS spectrophotometer (UV-1800, Shimadzu, Kyoto, Japan) at 420 nm, and P content was expressed as per cent.

##### 2.2.2.4 Potassium content (%)

Potassium content in the triacid digest was determined using the flame photometer Elico CL 361 (Piper, 1966) and expressed as per cent.

The nutrient uptake was computed using the formula:


$$\begin{array}{c} \text{N, P and K }\left({\text{kg ha}}^{-1}\right)\\ =\frac{\text{Nutrient content}\left(\%\right)\times \text{Dry matter }\left({\text{kg ha}}^{-1}\right)}{100} \end{array}$$

### 2.3 Data analysis

Analysis of variance (ANOVA) was estimated for individual environments, and further combined analysis of variance by split–split plot in completely randomized design was carried out using the software, Statistical Tool for Agricultural Research (STAR) (Version 2.0.1, http://bbi.irri.org/products) and R-Packages 1.5 (R Core Team 2013). If the F statistic is significant, further *post-hoc* pairwise comparison between sample means were computed using least significant difference (LSD) and Tukeys’ honest significant difference (HSD) test. The correlation coefficients and graphs were generated based on the mean data and correlograms were constructed in RStudio using “metan” package (Olivoto and Dal` Col Lucio, 2019) based on Pearson’s correlation matrix between soil chemical properties.

## 3 Results and discussion

### 3.1 Characterization

#### 3.1.1 X-ray diffraction characterization

**Figure 2:**
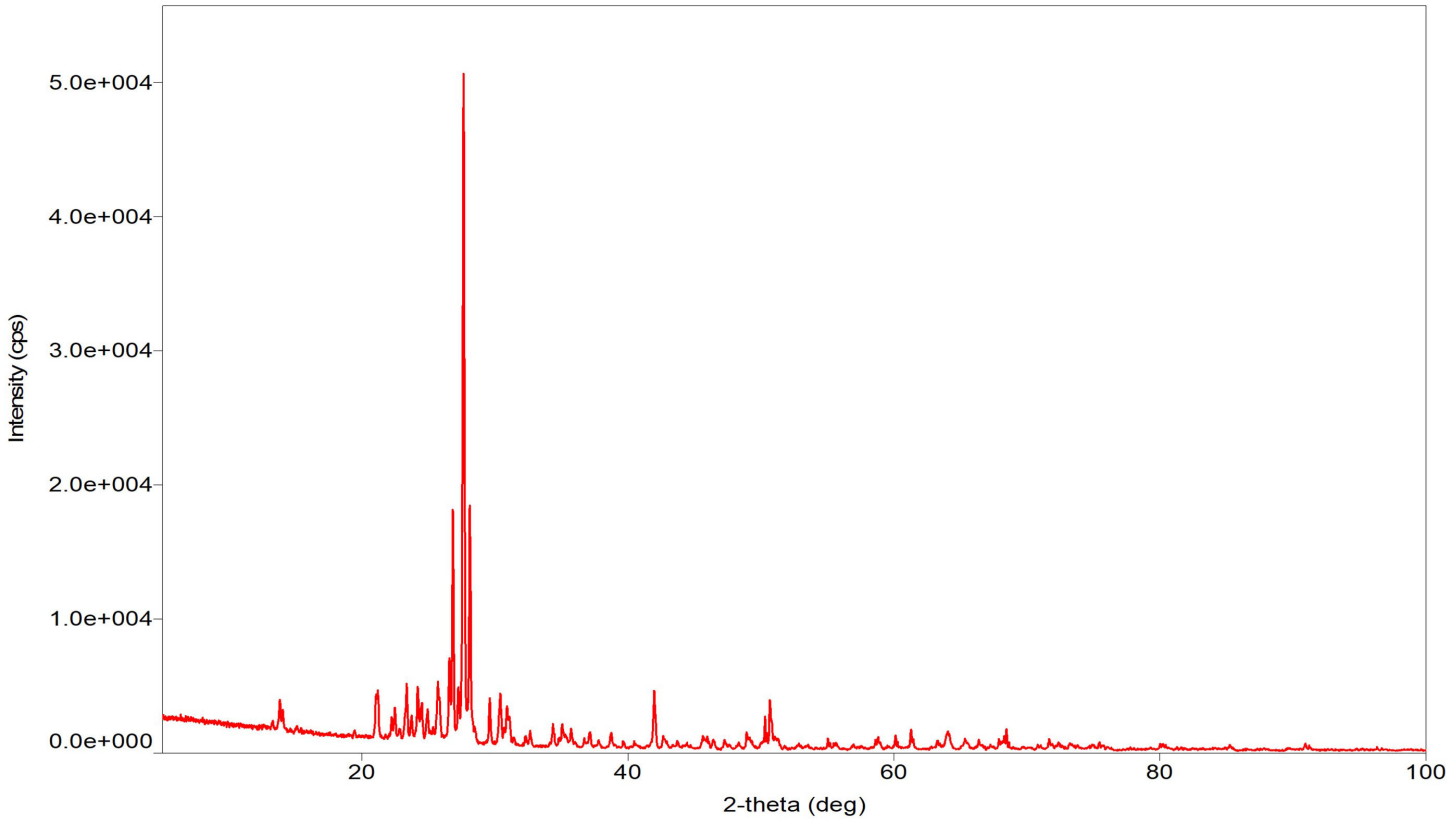
XRD pattern of zeolite sample.

X-ray diffraction (XRD) analysis was carried in order to identify the phases present in the zeolite sample as shown in **Figure 2**. XRD revealed the presence of mixture of phases in the collected zeolite with peaks at 2 that match mordenite, tetracesium tetraaluminosilicate, leucite, octasodium tecto hexagallohexa silicate dibromide, Ga-sodalite, lithosite, and cerium nickel silicon. The lattice parameters (a, b, c) obtained from the XRD for zeolite sample are shown in **Table 3**. Zeolites found in nature are rarely in their pure form but usually contain impurities such as other types of zeolite, other minerals, or amorphous materials. In this case, quartz impurities were found in mordenite as indicated by the XRD pattern. In our study, quantitative XRD analysis showed that the major component of the natural zeolite was mordenite with minor amounts of others. The diffraction pattern of the zeolite sample was matched with that of the literature, and identified phases are shown in **Table 3** along with the chemical formulas and figure of merit of the crystalline phases of zeolite (**Table 4**).

**Table 3:** Lattice parameters (a, b, c) of zeolite sample.

a(A)	b(A)	c(A)
16.75	13.68	5.08
20.27	17.80	7.52
12.89	12.89	13.51
9.01	9.01	9.01
15.23	10.25	8.45
23.56	4.26	13.85

**Table 4:** The details of crystalline phases of zeolite.

Phase name	Formula	Figure of merit
Zeolite CAS, tetracesium tetraaluminosilicate	Cs4 (Al4 Si20 O48)	3.047
Mordenite (Tm, Na)	Tm0.9 Na3.5 H0.8 Al7 Si41 O96 ·24 H2 O	2.961
Leucite	K (Al Si2 O6)	1.559
octasodium tecto-hexagallohexasilicate dibromide, Ga-Sodalite	Na8 (Ga Si O4)6 Br2	1.435
Lithosite	K3 (H Al2 Si4 O13)	1.716
Cerium nickel silicon	Ce7 Ni2 Si5	2.885

#### 3.1.2 Scanning electron microscopy

**Figure 3:**
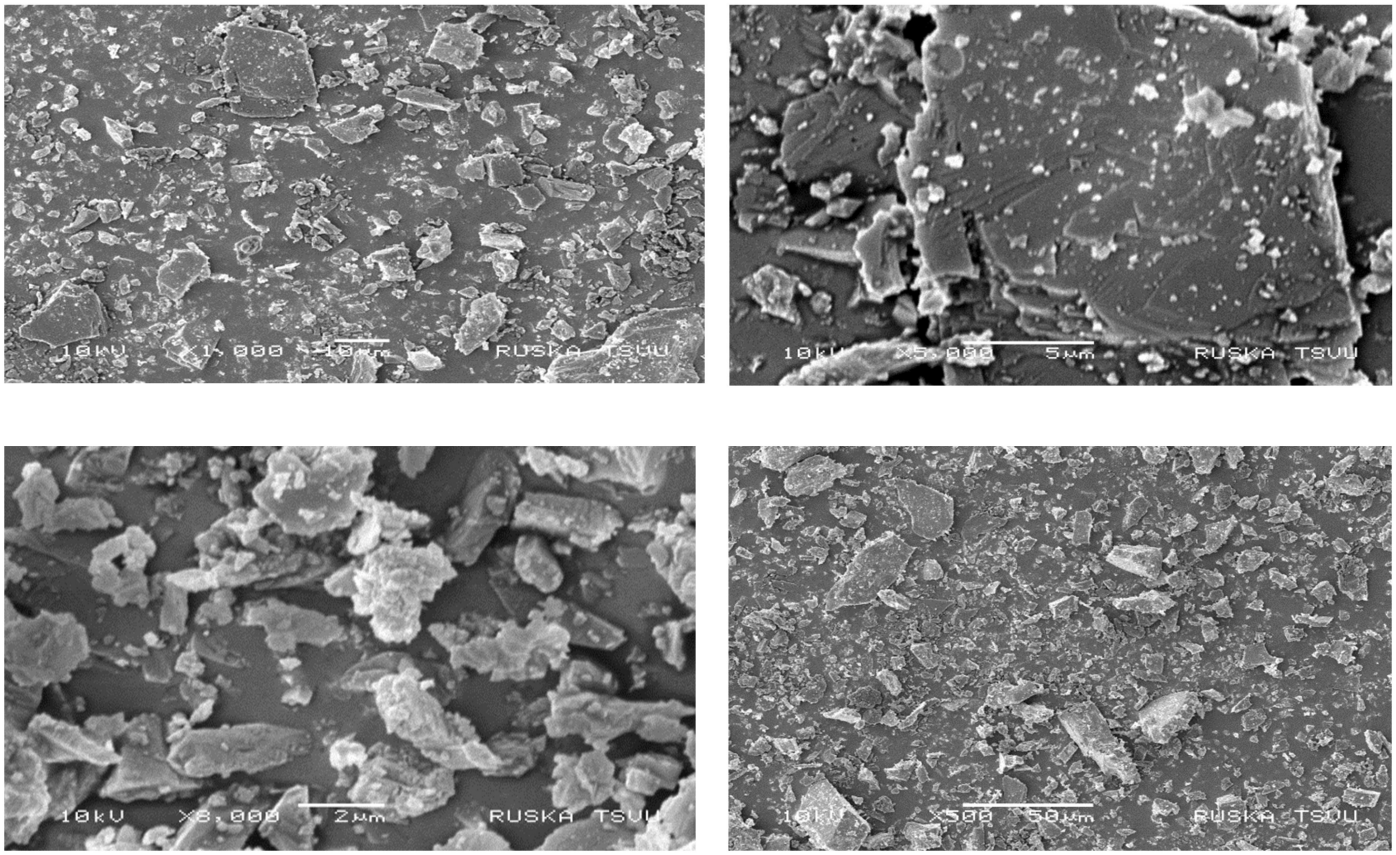
SEM images of zeolite captured at 2 µm, 5 µm, 10 µm, and 50 µm.

Scanning electron microscopy is a highly versatile technique that gives detailed surface information of samples and used to obtain high-resolution images. It is a type of electron microscopy that uses a focused beam of electrons to scan the surface of a specimen and generate images at a much greater resolution. The resolution of SEM instruments can range from up to several nanometres. With the help of scanning electron microscopy (SEM) imaging, the shape of zeolite was elucidated. In our study, the photographs at several thousands’ magnification have shown a needle-like structure (**Figure 3**). According to the reports on natural zeolites, the morphology of natural mordenite with a small amount of impurities was needle- or fibre-like with c-axis elongation (Passaglia and Sheppard, 2001). The SEM results are consistent with the XRD results, suggesting that the sample is mordenite zeolite. The SEM images were matching the images presented by Sakizci and Kilinc (2015).

### 3.2 Batch sorption studies with zeolite

**Figure 4:**
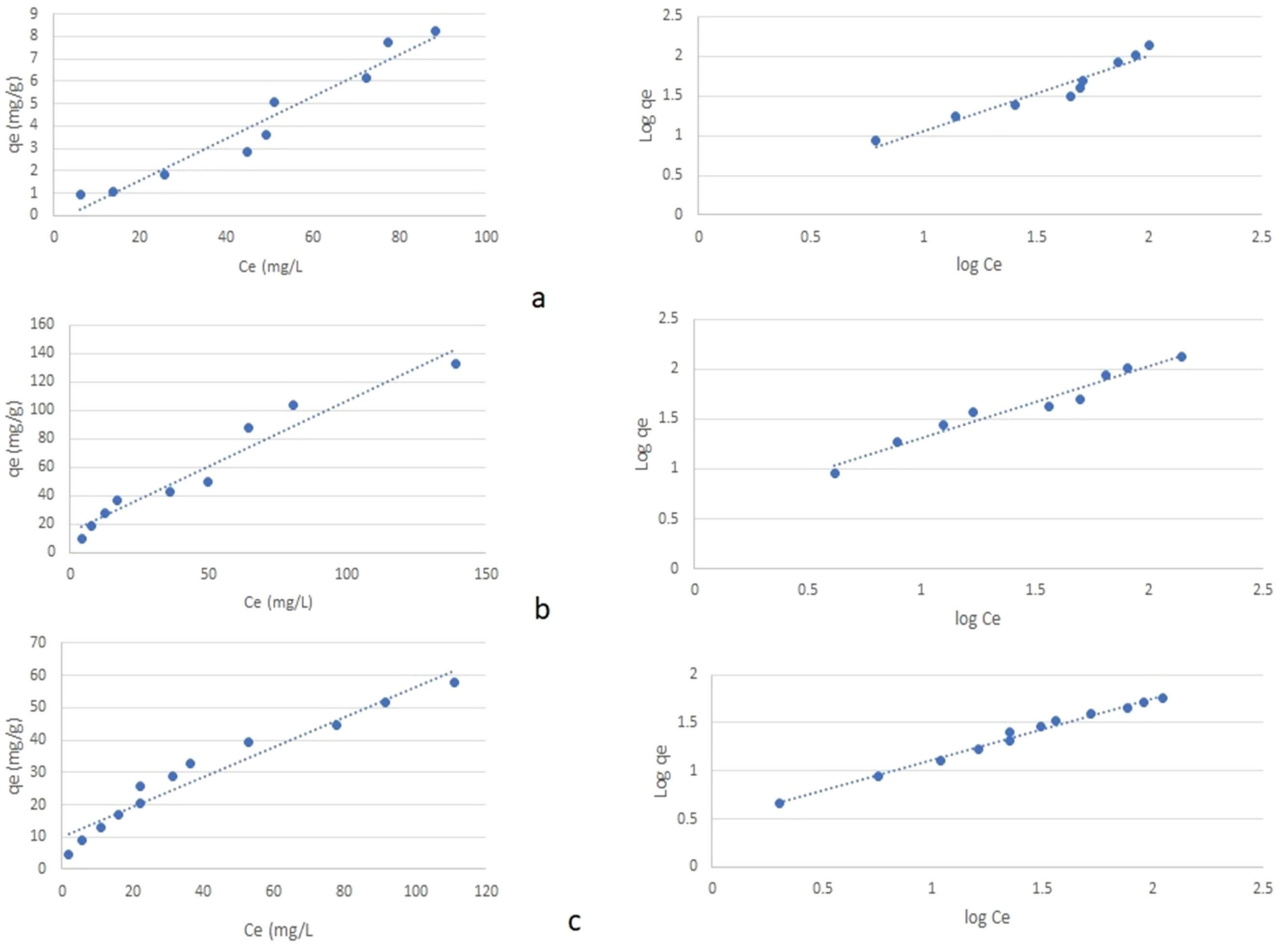
Isotherm plots (qe vs. Ce) and (log qe vs. log Ce) for adsorption of the ions **(A)** NH_4_^+^, **(B)** P, and **(C)** K on the natural zeolite.

Plant growth is mainly dependent on nutrients in soil solution and associated with colloid surfaces. In general, adsorbed fractions will be released slowly as the ions in soil solution get depleted. Thus, removal of ammonium nitrogen (NH_4_^+^–N), phosphorus (P), and potassium (K) by zeolite is important to know the nutrient supplying capacity. Therefore, this study was conducted to determine NH_4_^+^, P, and K adsorption characteristics of mordenite natural zeolite at high ionic concentrations (0–800 mg L^−1^) in aqueous solution. Results showed that initial NH_4_^+^, P, and K concentration had significant effects on NH_4_^+^, P, and K adsorption capacity of zeolite. With the addition of increasing amounts of ammonium (N), phosphorus (P), and potassium (K) to zeolite, the equilibrium concentration of N, P, and K increased (**Figure 4**). Similarly, with the increasing equilibrium concentration (Ce), an increase in the adsorption of N, P, and K was reported by Shin et al. (2021); Assawasaengrat and Rueangdechawiwat (2019), and Cheng et al. (2017) where the extent of adsorption varies with zeolite type and other conditions. The presence of cages and channels contain cations (usually Na^+^, Ca^2+^, K^+^, and Mg^2+^) and water molecules within the zeolite matrix will get exchanged with the cations presence in the surroundings. Hence, with supplementation of ammonium/potassium, NH_4_^+^-N/K gets exchanged with the cations present in its framework in zeolite in that it helps in nutrient supply to plants. Several factors like the negative charge of its framework structure, concentration, size, and charge of the exchange ions determine the exchange capacity of the zeolite. The extent of exchange capacity needs to be explored for different types of zeolite. Extensive literature is available with respect to ammonium exchange capacity of different zeolites in waste water. Further studies revealed that chemical modification of natural zeolite/clay has increased ammonium exchange capacity (AEC) to the extent of 45–55 g NH_4_^+^–N kg^−1^ (Guida et al., 2020). Zeolites can be modified with different treatments such as thermal treatment, acid treatment, alkaline treatment, surfactant, and even with others. The choice of the treatment depends on the purpose for which it is used; mainly chemical treatment with acids, bases, and salts enhances cation absorption and also is very simple, fast, and accessible. Chemical modification with NaOH, FeCl_3_, and HCl in clinoptilolite zeolite resulted in an increase in ammonium adsorption by an order of magnitude (Jahani et al., 2023).

In our study, adsorption data were fitted to sorption isotherms, Freundlich and Langmuir models, and found that all the three models fitted well with the adsorption process of the studied zeolite; however, the Langmuir model showed best fit for the adsorption process for NH_4_^+^–N while Freundlich for P and K (**Tables 5**, **6**). The maximum adsorption capacity of the studied zeolite for NH_4_^+^, P, and K were estimated as 10.6 mg g^−1^, 1.08 mg g^−1^, and 2.15 mg g^−1^, respectively. This might be attributed to the highest pore surface area and CEC. Wijesinghe et al. (2016) reported that natural Australian zeolites showed maximum adsorption capacity of NH_4_^+^ to the extent of 9.48 mg N g^−1^and 11.83 mg N g^−1^, respectively. Xue et al. (2018) employed different types of zeolites in ammonia-rich water to evaluate the ammonia removal efficiency and concluded that mordenite is more efficient in removing ammonia from water. The amount of NH_4_^+^ ions removed by zeolite from aqueous solutions increased with increasing concentrations of NH_4_^+^ ions in the purified solution (Franus et al., 2015). This suggests that the studied zeolite can be used for improving nutrient use efficiency of chemical fertilisers for crop production.

**Table 5:** Linear forms of Langmuir equations and constants b and k for N, P, and K adsorption by zeolite.

Sample	Equation	K* (mmol L^-1^)	b* (mmol kg^−1^)	R^2^
Zeolite/NH_4_^+^	Y=0.0942x−0.3228	0.291	10.62	0.95
Zeolite/P	Y=0.9257x+14.092	0.066	1.08	0.94
Zeolite/K	Y=0.4641x+10.095	0.046	2.15	0.94

K*= slope/intercept; b*= 1/slope.

**Table 6:** Freundlich equations and constants for N, P, and K sorption by zeolite.

Sample	Equation	n (kg L^−1^)	K_f_ (mmol kg^−1^)	R^2^
Zeolite/NH_4_^+^	Y=0.939x+0.1260	1.06	1.336	0.92
Zeolite/P	Y=0.719x+0.5915	1.39	3.90	0.96
Zeolite/K	Y=0.6397x+0.4739	1.56	2.97	0.99

Shin et al. (2021) reported that zeolite-based adsorbents showed the maximum K sorption to the extent of 40–42 mg g^−1^ by natural zeolite and treated zeolite, which showed higher K sorption rates than the natural zeolite. However, the maximum K sorption rate was found to be 2.15 mg g^−1^ in our study. At the lowest and highest concentration of NH_4_^+^–N, P, and K, a noticeable difference in the adsorption of ammonium, phosphorus, and potassium was reported in the zeolite. As compared to P, N and K were absorbed in large quantities by mordenite zeolite (**Tables 5**, **6**). The adsorption by zeolite followed the order: N>K>P. The adsorption maxima were also found to be high for ammonium followed by K and P (**Table 5**). The low adsorption maxima for P might be attributed to the negative surface on the zeolite. The surface charge for mordenite is −53 mV; hence, zeolite have poor affinity towards anions sorption. However, several studies state that surface modification of zeolite can increase affinity towards anions (Barczyk et al., 2014).

**Figure 5:**
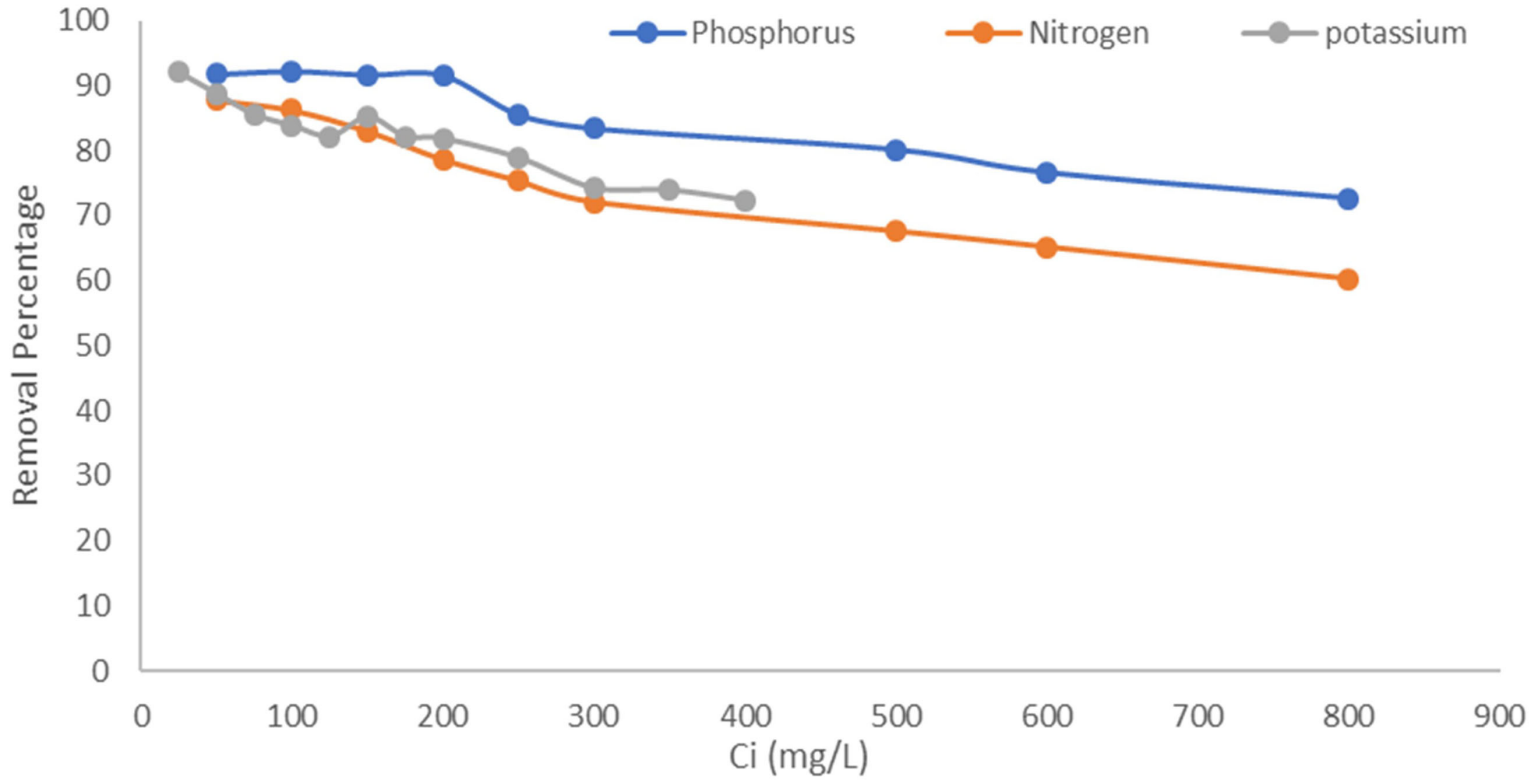
Removal capacity (%) of natural mordenite zeolite under different concentrations of N, P, and K.

The percentage of removal capacity ranges between 91% and 60% for ammonical N, P, and K (**Figure 5**). The removal efficiency is very high with low initial concentration, and as the concentration increased, there was a decline in removal capacity. Initially, the adsorption process is fast, as the ratio of the initial concentration of ammonia to the available surface area is low, and subsequently, the fraction adsorption increases. However, availability of active sites becomes less at higher concentration; hence, the percentage removal decreases. This observation is similar with phosphorus and potassium. As the ammonium concentration and potassium concentration increased, the removal capacity decreased. A similar phenomenon has also been observed in other studies (Kotoulas et al., 2019; Liu et al., 2022). Thus, initial ammonium concentration plays a key role in the adsorption mechanism of cations on zeolite. Sarioglu (2005) studied Dogantepe zeolite with initial ammonium concentration (8.8–885 mg NH_4_^+^–N L^−1^) and found that removal efficiency was achieved in the range of 8.8–40.3 mg NH_4_^+^–N L^−1^. Mordenite samples had an adsorption efficiency of approximately 8.7 mg total ammonia N g^− 1^ at a total ammonia N of 200 mg L^−1^ (Zhou et al., 2014), while Muscarella et al. (2023) revealed that the amount of NH_4_^+^ adsorbed by mordenite was high as compared to clinoptilolite and heulandite. This might be due to the high specific area of mordenite as compared to clinoptilolite and heulandite that allowed it to hold more of NH_4_^+^ ions. In addition, the higher the NH_4_^+^–N concentration in the solution, the higher the solute concentration gradient. This provides the necessary driving force so that NH_4_^+^–N ions could take the place of cations on the surface of the internal micropores of zeolite within a given contact time (Du et al., 2005; Cyrus and Reddy, 2011; Chai et al., 2021). In this study, experimental results clearly showed that zeolite used in the study can be a suitable adsorbent for the removal of NH_4_^+^ ion from aqueous solution, thus can be used to manage nitrogen availability in soils.

### 3.3 Column study

**Figure 6:**
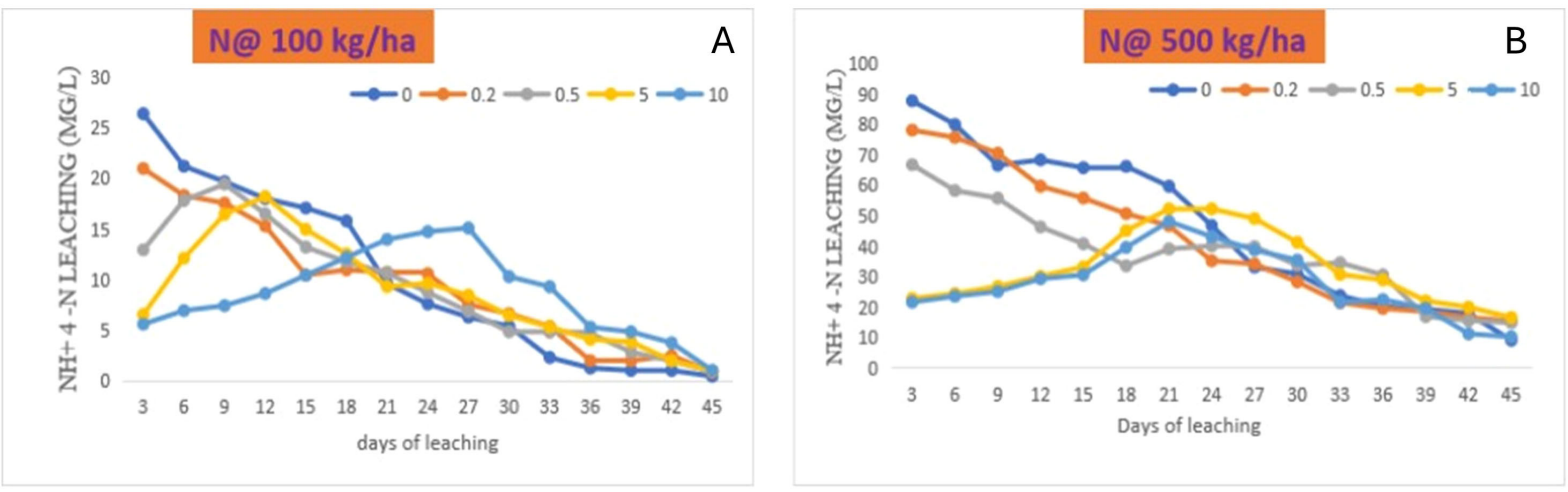
Effects of co-application of N though urea [**(A)** N @100 kg/ha, **(B)** N @500 kg/ha] and zeolite on NH_4_–N leaching at different leaching intervals in Alfisol soil.

Leaching experiment depicts the behaviour of nutrients in soil–water environment in the presence of fertiliser and zeolite. The effects of zeolite and N on ammonical-N leaching at different leaching events in Alfisol were compared with the combination of N with zeolite and without zeolite addition (**Figure 6**). The amount of NH_4_–N in leachate was largest in soil treated with N (supplied through urea), as compared to that of all the other treatments from day 3, and it continued to rise in the subsequent leaching events. Leachate ammonium concentration reached to maximum at day 12 with N @ 100 kg ha^−1^ + zeolite @ 5 tonnes ha^−1^, while the peak occurred at day 27 with the increase in zeolite application to 10 tonnes ha^−1^, and the concentration showed a decreasing trend afterwards, regardless of the rate of application (**Figure 6A**). Similarly, with N @ 500 kg ha^−1^, the NH_4_–N concentration was higher at day 3 with sole N supplied through urea as compared with N and zeolite application (**Figure 6B**). However, peak occurred at day 27 with zeolite application @ 10 tonnes ha^−1^ and followed a decreasing trend. The zeolite application could reduce the NH_4_–N losses when applied along with N as compared to sole N application.

**Figure 7:**
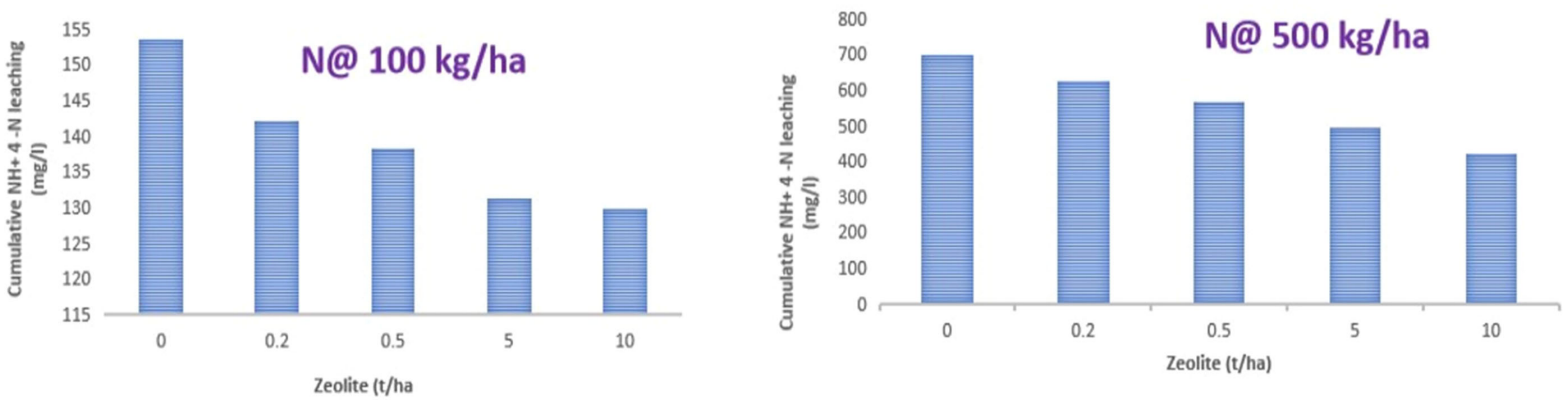
Effect of zeolite dose at two rates of urea application on cumulative losses of NH_4_–N from Alfisol soil. Means with different letters indicate significant differences among treatments by Tukey’s test at *p* ≤ 0.05.

During this experiment, cumulative amounts of NH_4_^+^–N measured in leachate is shown in **Figure 7**. In this study, the maximum NH_4_^+^–N was lost with N application without zeolite addition in both the case of N application rate @ 100 kg ha^−1^ and 500 kg ha^−1^. However, a significant reduction in the loss of NH_4_–N was observed with the increase in the rate of zeolite application. Similarly, the extent of reduction in the loss of NH_4_–N was high with N application rate of 500 kg ha^−1^ as compared to 100 kg ha^−1^ for Alfisol soil. With application of N @ 500 kg ha^−1^ + zeolite @ 10 tonnes ha^−1^ could reduce the loss of NH_4_–N up to 39.6%, while the application of N @ 100 kg ha^−1^ + zeolite @ 10 tonnes ha^−1^ could reduce the loss of NH_4_–N up to 15.4% as compared to the sole application of N @ 500 kg ha^−1^ and 100 kg ha^−1^, respectively. Therefore, zeolite application @ 10 tonnes ha^−1^ proved to be the most effective treatment in reducing leaching of NH_4_–N in Alfisol soil. This could be attributed to the specific selectivity of mordenite zeolite for NH_4_–N as evident from our sorption study; the mordenite zeolite could successfully reduce the leaching losses that in turn depends on the inner channels of zeolite. Upon evaluating the capacity of two natural New Zealand zeolites (clinoptilolite and mordenite) in removing NH_4_^+^ from a range of wastewaters, it was found that both zeolites tested, regardless of their particle sizes, were equally effective (87%–98%) at NH_4_^+^ removal from domestic wastewaters or synthetic solutions containing NH_4_^+^ concentrations of up to 150 g NH_4_–N m^−3^. However, mordenite showed more effective NH_4_^+^ removal than clinoptilolite for dairy and piggery wastewaters, and for synthetic solutions containing high NH_4_^+^ concentrations (350-750 g NH_4_–N m^−3^) (Nguyen and Tanner, 1998). In the leaching column experiment study with clinoptilolite zeolite, the leaching of ammonia from urea significantly reduced compared to sole soil alone (Latifah et al., 2017). Colombani et al. (2015) found a high concentration of N content in the leachate, while N content was low and retarded peaks were found in case of amended soil columns. In another study, the application of zeolite @ 10 tonnes ha^−1^ reduced the loss of nitrate-N, ammonical-N, and total-N by 19.4%, 16.9%, and 16%, respectively, in the leachate (Wang et al., 2023).

### 3.4 Pot experiment

#### 3.3.1 Effects of zeolite amendment with chemical fertilisers on tomato growth

##### 3.3.1.1 *Number of leaves and* p*lant height*

**Figure 8:**
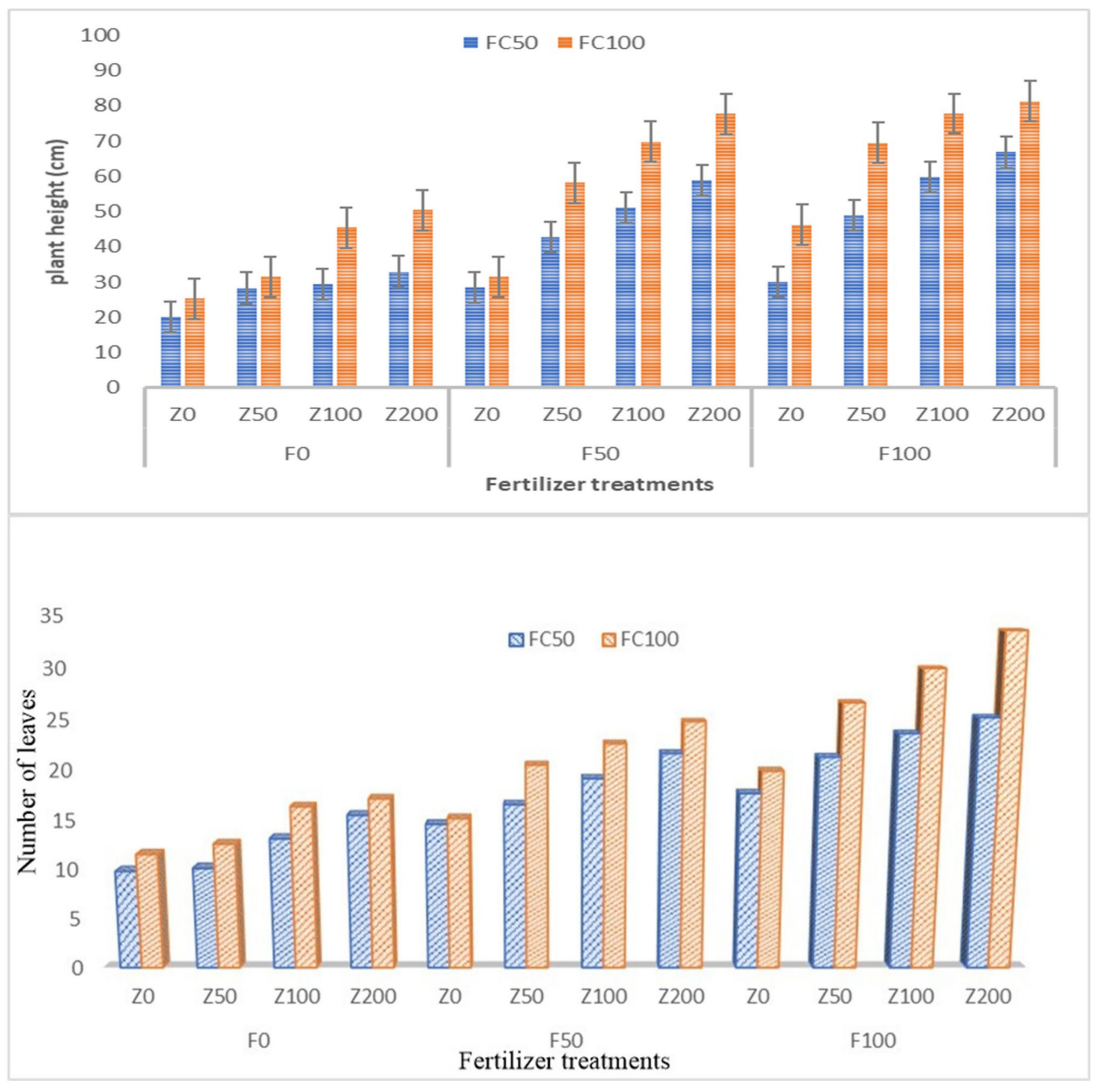
Impact of zeolite application along with fertilisers on **(A)** plant height and **(B)** number of leaves in tomato.

In our study, it is clear that zeolite application could maintain a greater number of leaves in combination with fertiliser application as compared to sole application of fertilisers (**Figure 8**). Without fertiliser addition, the zeolite application alone could not record the highest number of leaves in tomato. This is because zeolite as such does not contain nutrients to adequate levels. It can act as good exchanger and improve the nutrient content of fertilisers added to the soil (Jarosz et al., 2022). The mean values ± margin of error of plant height in tomato are presented in **Figure 8**. In our study, zeolite application along with fertilisers showed positive influence on the tomato plant height in both the moisture levels (50% FC and 100% FC). The plant height was high when grown under 100% FC with mean of 55.65 cm as compared to 50% FC with mean of 41.45 cm. With increase in zeolite levels, there was an increase in plant height with highest application of zeolite @ 200 kg ha^−1^ (Z200). In both the moisture levels, it was found that with RDF100 in combination with Z200, it could maintain highest plant height as compared to treatment combinations. Wu et al. (2020) observed that the application of 10 tonnes ha^−1^ zeolite improved grain filling in rice with increased amount of N accumulation. Hazrati et al. (2017) reported that zeolite application could alleviate water stress and other adverse effects and improved plant height and number of leaves in *Aloe vera* L. This finding was similar in rice (Wulandari et al., 2019) and maize grown in saline conditions (Aboul-Magd et al., 2020).

#### 3.3.2 Effect of zeolite amendment with chemical fertilisers on soil properties

**Figure 9:**
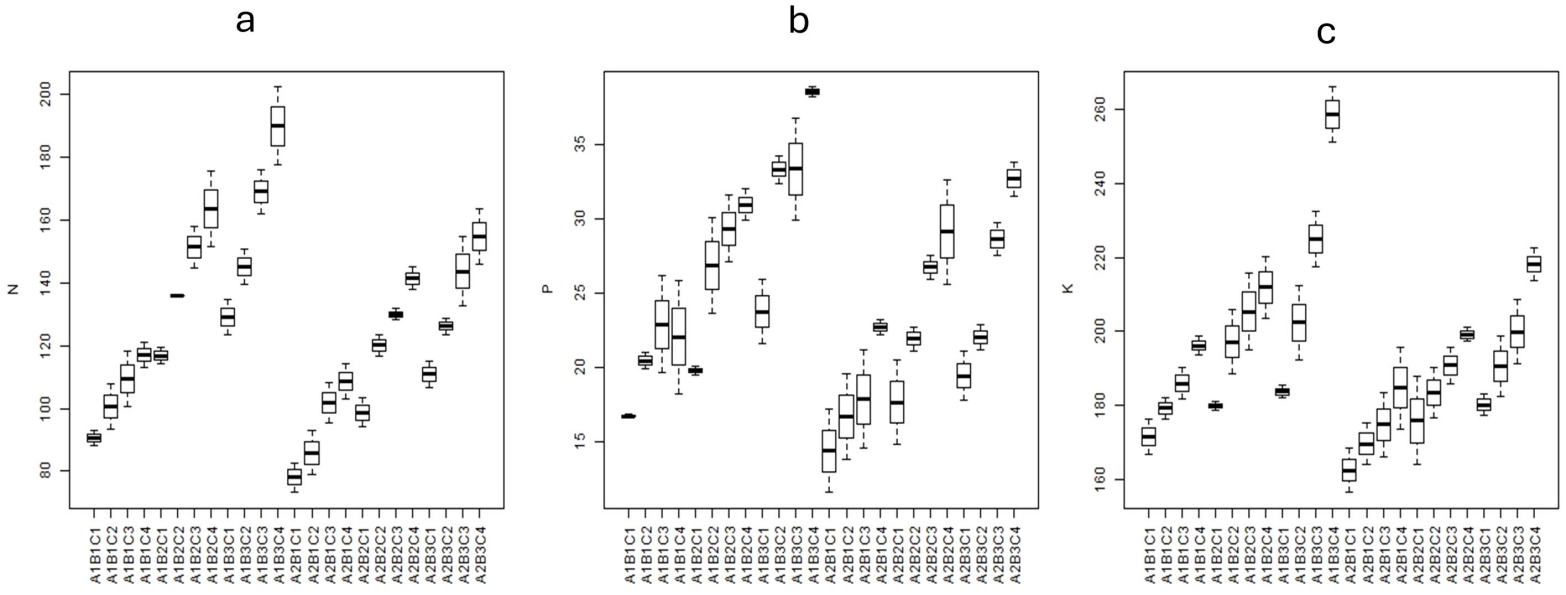
Box plots showing the **(A)** soil available N, **(B)** soil available P, and **(C)** soil available K content (kg/ha) in different treatments. A1 = 100% FC; A2 = 50% FC; B1 = control, B2 = 90: 50: 25 kg NPK/ha, B3 = 180: 100: 50 kg NPK/ha; C1 = control; C2 = 50 kg/ha; C3 = 100 kg/ha; C4 = 200 kg/ha.

At the end of each experimental period, soil available N, P, and K was studied in post-harvest soil samples. The change in soil available nitrogen content of the post-harvest soil at different moisture levels, fertiliser levels, and zeolite levels is presented in **Table 7**. There was a significant effect (p<0.05%) on the soil available N, P, and K due to the application of zeolite at both the moisture levels and fertiliser levels. The results revealed that fertiliser application along with zeolite application significantly improved soil available N, P, and K content (**Table 7**). Under two moisture conditions, it was observed that soil available N, P, and K content increased significantly under 100% FC as compared to 50% FC, while under three fertiliser levels studied, 100% RDF could maintain higher soil available N, P, and K content as compared to 50% RDF and control. The soil available N increased with increasing dose of zeolite application. Highest was observed with zeolite application (Z200) of 89.28 mg kg^−1^ (equivalent to 200 kg zeolite ha^−1^). The treatment RDF100 + Z4 was most effective in maintaining higher soil available nutrient under both the moisture levels, and the increase in soil available N, P, and K was significantly (p < 0.05%) higher than the rest of the treatments. This suggests that zeolite application improves soil available nutrients if applied along with chemical fertilisers. Legese et al. (2024) impregnated zeolite (phillipsite) with 5% solutions of macro- (P, N, K, Ca, and Mg) and micro-nutrients (Fe, Zn, and Cu) in the form of their salts and evaluated the two samples (UNZC and SMNZC); it was found that total N increased in all amended plots as compared to amended plots. The content was higher where a higher dose was tested. Ravali et al. (2020) observed similar findings due to the application of zeolite @ 7.5 tonnes ha^−1^ and N @ 200 kg ha^−1^, which showed a significant effect on soil NPK content at different crop growth stages and at harvest in maize. Under 100% FC, with 100% RDF, the application of zeolite @ 200 kg ha^−1^, 100 kg ha^−1^, and 50 kg ha^−1^ increased the soil N by 48.6%, 40.5%, and 25.1%, respectively, over Z0 (without zeolite application) (**Figure 9A**). With 50% RDF, the application of zeolite @ 200 kg ha^−1^, 100 kg ha^−1^, and 50 kg ha^−1^ increased the soil N by 62.1%, 44.7%, and 34.9%, respectively, over Z0. Under 50% FC, with 100% RDF, the application of zeolite @ 200 kg ha^−1^, 100 kg ha^−1^, and 50 kg ha^−1^ increased the soil N by 37.5%, 27.8%, and 22.2%, respectively, over Z0 (without zeolite application). With 50% RDF, the application of zeolite @ 200 kg ha^−1^, 100 kg ha^−1^, and 50 kg ha^−1^ increased the soil N by 31.5%, 24.7%, and 4.7%, respectively, over Z0. Zeolite application @ 5 tonnes ha^−1^ resulted in an increase of 35.13% in total N content in Alfisols (Triatmoko et al., 2019). The soil chemical properties that include soil cation exchange capacity, the soil total nitrogen concentration, and nitrogen use efficiency increased significantly due to the co-application of 150 kg N ha^−1^ and 200 kg N ha^−1^ with 10 and 15 tonnes ha^−1^ of zeolite (Dastbaz et al., 2023).

**Table 7:** Soil available N, P, and K (kg/ha) as affected by different levels of moisture, fertiliser, and zeolite.

		Soil available N					
		FC (100%)			FC (50%)		
		Control	50% RDF	100%RDF	control	50% RDF	100%RDF
2017	Z0	93.19c	119.49d	134.78d	82.76b	103.55c	114.98c
	Z50	107.67b	135.97c	150.79c	93.10b	123.65b	128.78b
	Z100	118.45ab	158.08b	175.89b	108.43a	131.73b	154.74a
	Z200	121.07a	175.55a	202.24a	114.34a	144.96a	163.66a
2018	Z0	88.15c	114.11c	123.32d	73.30b	94.03c	106.71c
	Z50	93.37bc	136.05b	139.39c	78.80b	116.51b	123.61b
	Z100	100.67b	144.63ab	162.11b	95.58a	128.16a	132.55b
	Z200	112.84a	151.43a	177.39a	103.23a	137.75a	145.97a
	Interaction effect	MxF	MxZ	FxZ	MxFxZ	YxMxFxZ	
		**	**	**	**	**	

		Soil available P					
		Control	50% RDF	100%RDF	Control	50% RDF	100%RDF
2017	Z0	16.81c	20.07b	25.87c	17.17b	20.45c	21.04c
	Z50	20.98b	30.07a	34.22b	19.57ab	22.71c	22.88c
	Z100	26.12a	31.54a	36.76ab	21.12a	27.51b	29.73b
	Z200	25.83a	31.96a	38.14a	22.20a	32.63a	33.80a
2018	Z0	16.59a	19.49c	21.59c	11.58b	14.81c	17.76c
	Z50	19.89a	23.65b	32.37b	13.83b	21.11b	21.16c
	Z100	19.63a	27.08ab	29.85b	14.56b	25.90a	27.50b
	Z200	18.23a	29.84a	38.81a	23.16a	25.55a	31.58a
	Interaction effect	MxF	MxZ	FxZ	MxFxZ	YxMxFxZ	
		**	**	**	**	**	

		Soil available K					
		Control	50% RDF	100%RDF	Control	50% RDF	100%RDF
2017	Z0	166.91c	178.91c	185.47c	156.59b	164.06c	177.27c
	Z50	176.34bc	188.72bc	192.44c	164.12ab	176.66bc	182.37bc
	Z100	181.67ab	194.92ab	217.38b	166.34ab	186.03ab	191.39b
	Z200	193.61a	203.66a	265.97a	173.77a	197.28a	213.85a
2018	Z0	176.25c	181.98c	182.15d	168.52c	187.92b	183.11d
	Z50	182.26bc	205.82b	212.45c	175.23bc	190.38b	198.70c
	Z100	190.41ab	215.83a	232.50b	183.43b	195.60ab	208.54b
	Z200	198.81a	220.27a	251.26a	195.74a	201.14a	222.48a
	Interaction effect	MxF	MxZ	FxZ	MxFxZ	YxMxFxZ	
		**	**	**	**	**	

The same lowercase letters in each row indicate no significant differences by Tukey’s HSD test for α=0.05.

** significances levels.

Similarly, under 100% FC, with 100% RDF, the application of zeolite @ 200 kg ha^−1^, 100 kg ha^−1^, and 50 kg ha^−1^ increased the soil available P by 48.9%, 27.1%, and 23.7%, respectively, over Z0 (without zeolite application). With 50% RDF, the application of zeolite @ 200 kg ha^−1^, 100 kg ha^−1^, and 50 kg ha^−1^ increased the soil N by 37.8%, 28.4%, and 17.9% respectively, over Z0 (**Figure 9B**). Under 50% FC, with 100% RDF, the application of zeolite @ 200 kg ha^−1^, 100 kg ha^−1^, and 50 kg ha^−1^ increased the soil N by 44.9%, 29.5%, and 11.6%, respectively, over Z0 (without zeolite application). With 50% RDF, the application of zeolite @ 200 kg ha^−1^, 100 kg ha^−1^, and 50 kg ha^−1^ increased the soil N by 43.9%, 42.1%, and 26.4%, respectively, over Z0. The capacity of zeolite to improve the P availability is found high under 100% FC as compared to 50% FC.

In case of soil available K, under 100% FC, with 100% RDF, the application of zeolite @ 200 kg ha^−1^, 100 kg ha^−1^, and 50 kg ha^−1^ increased the soil available K by 14.1%, 12.4%, and 6.92%, respectively, over Z0 (without zeolite application) (**Figure 9C**). While with 50% RDF, the application of zeolite @ 200 kg ha^−1^, 100 kg ha^−1^, and 50 kg ha^−1^ increased the soil N by 14.6%, 10.6%, and 6.97%, respectively, over Z0. Under 50% FC, with 100% RDF, the application of zeolite @ 200 kg ha^−1^, 100 kg ha^−1^, and 50 kg ha^−1^ increased the soil N by 21.1%, 15.8%, and 7.53%, respectively, over Z0 (without zeolite application). While with 50% RDF, the application of zeolite @ 200 kg ha^−1^, 100 kg ha^−1^, and 50 kg ha^−1^ increased the soil N by 21.5%, 19.1%, and 7.01%, respectively, over Z0. The capacity of zeolite to improve the nutrient availability is found high under 100% FC as compared to 50% FC. There was significant difference in soil available nutrients in post-harvest soil due to zeolite and fertiliser use. The combined application of zeolite and fertiliser showed improved nutrient status than fertiliser application without zeolite. Some studies reported that N fertiliser requirement was reduced by 33% due to zeolite application in rice (Chen et al., 2017). The two- and three-way interaction effect among moisture, fertiliser rate, zeolite, and even year was significant for soil available N, P, and K contents. Averaged across the three zeolite amendments, topsoil available K increased from 13% (93 DAT, K_30_) to 31% (55 DAT, K_60_) in 2017, and from 16% (55 DAT, K_30_) to 46% (34 DAT, K_60_) in 2018 due to K_30_ and K_60_ applications application in rice (Li et al., 2022). In calcareous soils, it was reported that application of natural zeolite at 10 μg ha^−1^ (Z10) significantly increased in N, P, and K in post-harvest soil samples with 42.72 mg N kg^−1^ soil, 20.23 mg P kg^−1^ soil, and 201.05 mg K kg^−1^ soil, respectively (Saleh and Al Bahrani, 2023). Application of zeolite along with chemical fertilisers could improve soil N, P, and K content (Banik et al., 2023) and also with organic manures such as vermicompost and biofertilisers, and chemical fertilisers could improve soil N, P, and K content and also resulted in higher soil available N, P, and K in post-harvest soil samples (Rahmani et al., 2023; Idim et al., 2024).

#### 3.3.3 Effects of zeolite amendment with chemical fertilisers on N, P, and K uptake in tomato

**Figure 10:**
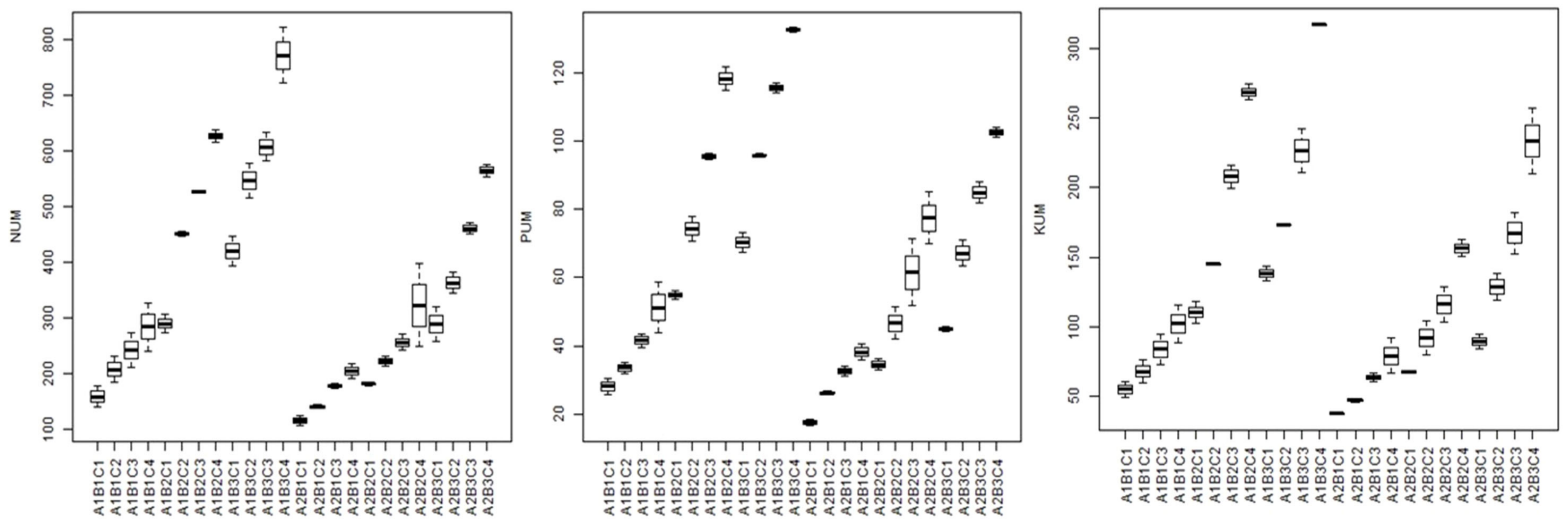
Box plots showing the N, P, and K uptake (mg/pot) in different treatments for both the study years. NUM, nitrogen uptake; PUM, phosphorus uptake; KUM, potassium uptake; A1 = 100% FC; A2 = 50% FC; B1 = control, B2 = 90: 50: 25 kg NPK/ha, B3 = 180: 100: 50 kg NPK/ha; C1 = control; C2 = 50 kg/ha; C3 = 100 kg/ha; C4 = 200 kg/ha.

The present study showed higher N, P, and K uptake due to zeolite addition along with chemical fertilisers (**Figure 10**). Experimental treatments significantly affected N, P, and K uptake in tomato. Among the treatments, control treatment (RDF0Z0) had minimum N, P, and K uptake, whereas maximum N, P, and K uptake was obtained due to application of full recommended dose of fertilisers along with 200 kg zeolite ha^−1^ (RDF100Z200). The zeolite addition improve,d N, P and K uptake by two times as compared to without zeolite addition under both the moisture conditions. The treatment RDF100+Z200 could maintain higher mean N, P, and K uptake with 560.63 mg pot^−1^, 85.34 mg pot^−1^, and 192.87 mg pot^−1^, respectively, in 100% FC conditions, while least mean N, P, and K uptake with 289.39 mg pot^−1^, 42.80 mg pot^−1^, and 83.30 mg pot^−1^, respectively, in 100% FC conditions in control treatment (RDF0Z0). However, the uptake reduced slightly under 50% FC conditions where the mean N, P, and K uptake was 436.53 mg pot^−1^, 87.53 mg pot^−1^, and 192.87 mg pot^−1^, respectively, in F100+Z200 treatment and 224.90 mg pot^−1^, 42.80 mg pot^−1^, and 76.63 mg pot^−1^, respectively, under RDF0Z0 treatment. Zeolite addition (5 tonnes ha^−1^) along with manures improved potassium availability and uptake in soybean cultivated in Alfisols (Haniati et al., 2019) and also improved N availability and uptake in soybean (Triatmoko et al., 2019). In Iran, there was significant effect of combined application of zeolite and fertiliser on improved N uptake (Kavoosi, 2007) and K uptake in rice (Chen et al., 2017; Xia et al., 2019). Gholamhoseini et al. (2012) reported that the zeolite application @ 9 tonnes ha^−1^ improved the N uptake by four times in canola.

#### 3.3.4 Effects of zeolite amendment with chemical fertilisers on tomato biomass and WUE

In the first year of the experiment (2017), zeolite addition to the soil (along with chemical fertiliser) significantly increased the tomato biomass in respect to the control (**Table 8**). The yield at the highest zeolite dose (Z200) was higher with 34.82 g pot^−1^ than other doses. This observation was similar in second year of experimentation (2018) where it was 39.99 g pot^−1^ due to application zeolite @ 200 kg ha^−1^ (Z200). Result showed that the biomass of tomato was significantly influenced by the treatment applied (**Table 8**). The greatest mean biomass yield was obtained by supplementing (RDF_100_Z_200_) under both moisture levels studied with 49.08 g pot^−1^ in 100% FC and 38.31 g pot^−1^ in 50% FC and the least with 26.93 g pot^−1^ in 100% FC and 26.06 g pot^−1^ in 50% FC in control (RDF_0_Z_0_). On other hand, with the increase in fertiliser rate from RDF0 to RdF100 (100% RDF-180: 100: 50 kg NPK ha^−1^) and zeolite dose from Z0 to Z200 (0–200 kg ha^−1^) led to an increase of 36.4% and 48.39% increase in biomass yield, respectively. Kotoulas et al. (2019) reported highest fresh weight of wheat due to zeolite application @ 40 mg kg^−1^, which was fund to be 74% higher than the control. Another study revealed that combined application of 100 kg ha^−1^ zeolite and 200 kg ha^−1^ NPK provided optimal yields of dry shelled maize up to 5.44 tonnes ha^−1^ grown in Inceptisol (Affendi et al., 2023).

**Table 8:** Dry matter yield (g/pot) and WUE (g L^−1^) as affected by different levels of moisture, fertiliser, and zeolite in tomato.

		Dry matter yield (g/pot)					
		FC (100%)			FC (50%)		
		Control	50% RDF	100%RDF	control	50% RDF	100%RDF
2017	Z1	22.60b	26.55c	30.73d	20.40c	24.26c	28.60d
	Z2	23.11b	30.06b	34.05c	22.42bc	26.40bc	31.19c
	Z3	25.53a	30.79b	37.33b	24.58ab	28.03b	35.06b
	Z4	26.93a	35.38a	49.08a	26.06a	33.13a	38.31a
2018	Z1	21.34c	30.87d	33.60d	19.94c	23.54d	26.49d
	Z2	23.30bc	37.57c	42.30c	22.31b	30.56c	31.23c
	Z3	25.24ab	41.09b	51.27b	24.44ab	35.60b	37.88b
	Z4	26.74a	44.17a	58.82a	26.44a	41.37a	42.39a

		WUE (g/L)					
		Control	50% RDF	100%RDF	control	50% RDF	100%RDF
2017	Z1	5.38b	6.32c	7.32d	9.71c	11.55d	13.62d
	Z2	5.50b	7.15b	8.11c	10.67b	12.57c	14.85c
	Z3	6.08ab	7.33b	8.88b	11.70a	13.35b	16.70b
	Z4	6.41a	8.42a	11.68a	12.40a	15.77a	18.24a
2018	Z1	5.08b	7.35c	8.00d	9.49d	11.21d	12.61d
	Z2	5.54ab	8.94b	10.07c	10.62c	14.55c	14.87c
	Z3	6.01a	9.78a	12.21b	11.64b	16.95b	18.04b
	Z4	6.36 a	10.51a	14.01a	12.59a	19.70a	20.19a

The same lowercase letters in each row indicate no significant differences by Tukey’s HSD test for α=0.05.

Zeolite addition to the soil (along with chemical fertiliser) significantly increased the WUE as compared to the control (**Table 8**). The WUE was found high with 50% FC as compared to 100% FC during both the years of experiment. The WUE was found high under 50% FC with 18.24 g L^−1^ in 2017 and 20.19 g L^−1^ in 2018 in F_100_Z_200_ treatment, while under 100% FC, the WUE was with 11.68 g L^−1^ in 2017 and 14.01 g L^−1^ in 2018 in F_100_Z_200_ treatment. Under 100% FC, the mean increase in WUE over 2 years with Z4 was 22.17% in control (without any fertiliser application), 38.11% in 50% RDF, and 67.34% in 100% RDF. Research states that zeolite holds water in its extraordinary porous structure and hence improves water availability to crops and enhances WUE (Xiubin and Zhanbin, 2001; Gholamhoseini et al., 2013). Zheng et al. (2018) reported that zeolite application @ 15 tonnes m^−2^ along with energy-controlled irrigation improved grain yield with less water consumption. According to Kralova et al. (1994), soils enriched with natural zeolite were able to increase the water retention by 18%–19% and for sandy soils even up to 50% (Lancellotti et al., 2014). While Ravali et al. (2020) reported 48.5% increase in water retention capacity due to 7.5 tonnes ha^−1^ zeolite application in sandy and loamy soils. Hazrati et al. (2022) reported an increase in WUE up to 22.10 g L^−1^ with the use of 10 tonnes zeolite ha^−1^ along with an application @ 150 kg ha^−1^ even under 60% depletion of available soil water content in *Salvia officinalis* grown in loam sandy soils. The positive effect of zeolite application in increasing yield was observed in maize (Aboul-Magd et al., 2020); sugarcane (Cairo et al., 2017), and tomato (Méndez Argüello et al., 2018).

#### 3.3.5 Correlation

**Figure 11:**
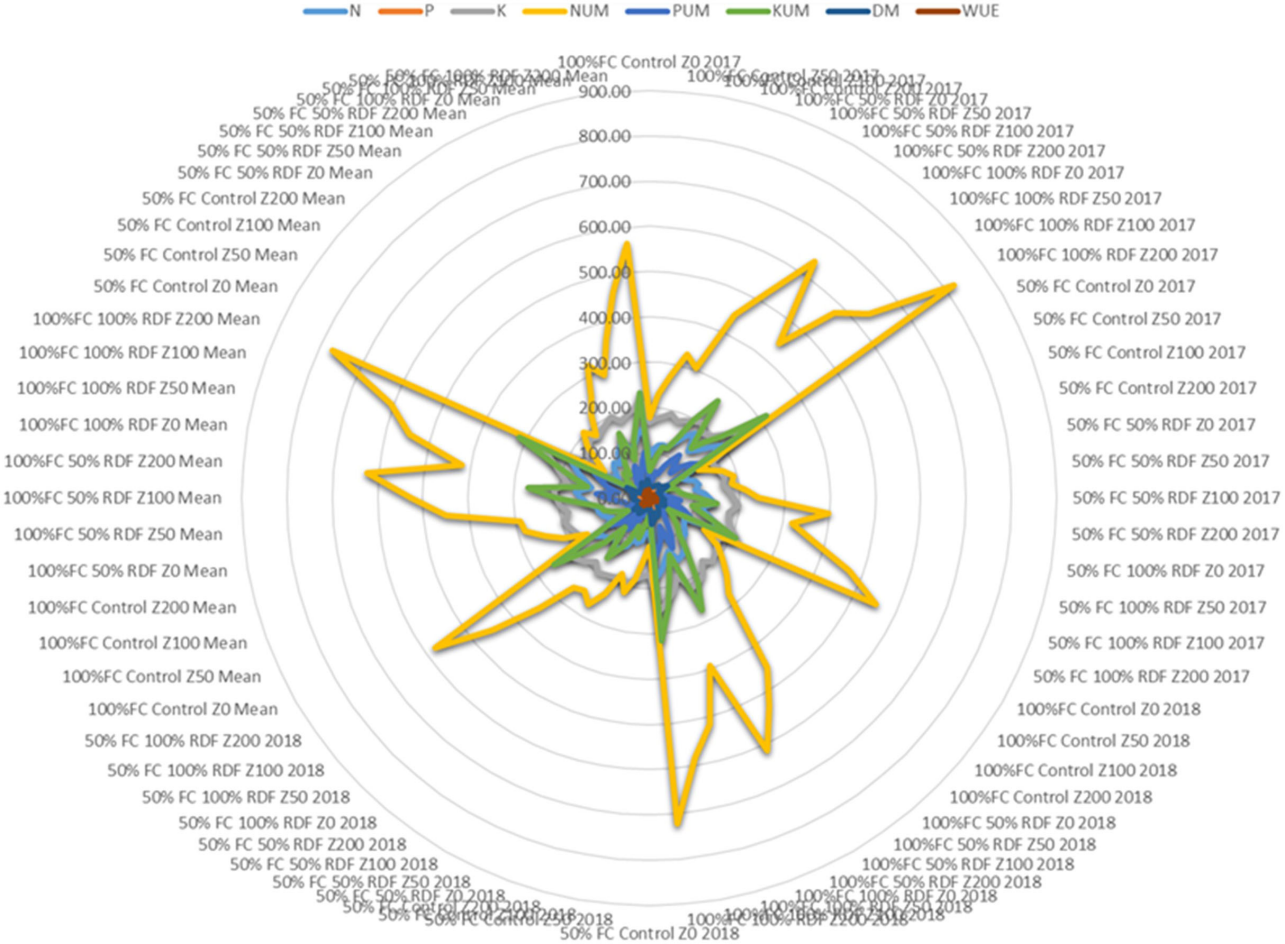
Spider diagram of different treatments on soil available N (denoted as N), P (denoted as P), K (denoted as K); plant uptake of N (denoted as NUM), plant uptake of P (denoted as PUM), plant uptake of K (denoted as KUM); dry matter production (denoted as DM) and water use efficiency (WUE).

**Figure 12:**
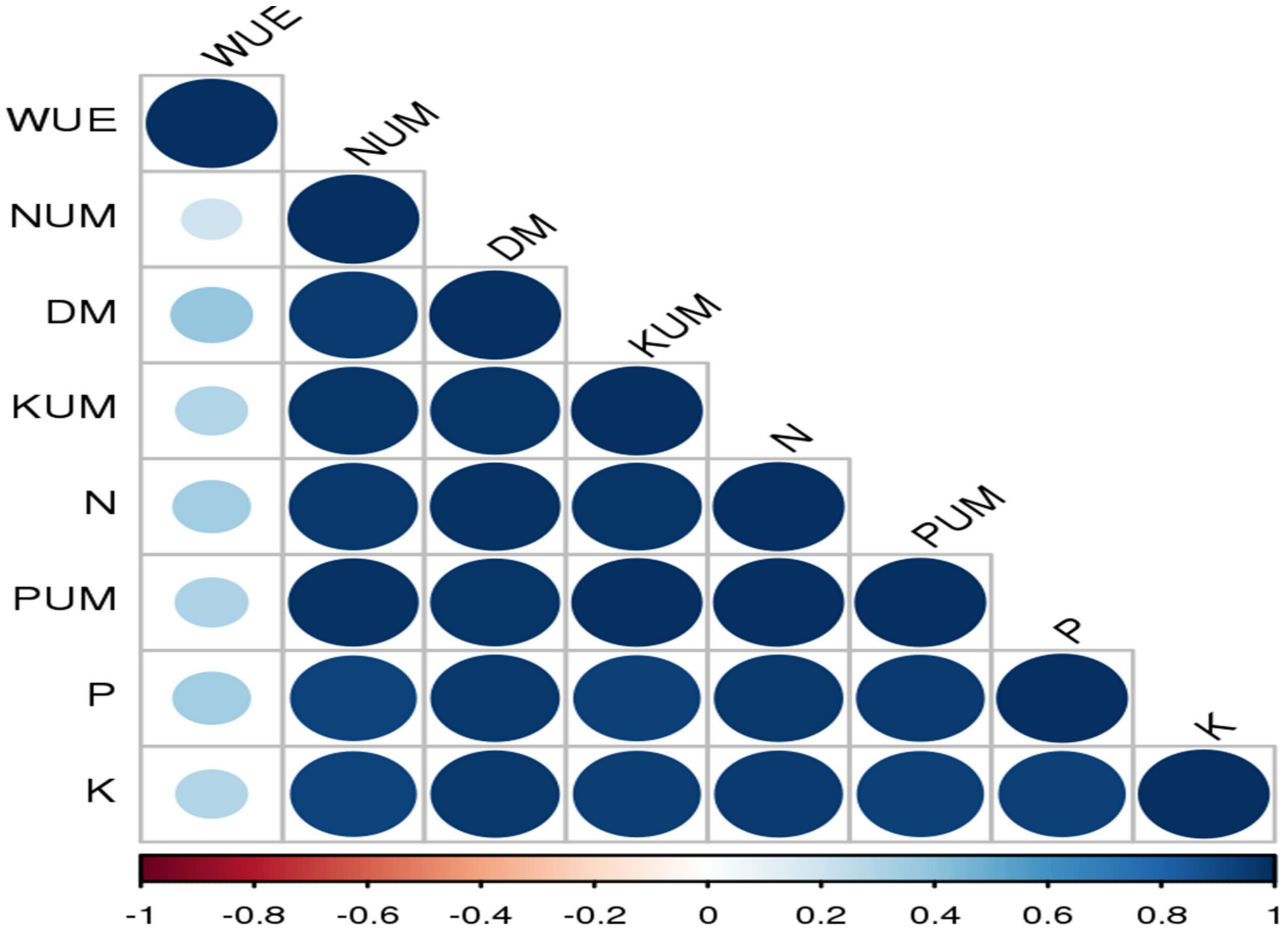
Pearson’s correlation matrix between soil chemical properties (soil available N, P, and K), nutrient uptake (NUM, PUM, and KUM), WUE, and dry matter yield. Correlations are displayed in blue (positive) and red (negative); colour intensity and circle size are proportional to the correlation coefficient. WUE, water use efficiency; NUM, N uptake; DM, dry matter; KUM, potassium uptake; N, soil available N; PUM, P uptake; P, soil available P; K, soil available K.

In most of the studies, clinoptilolite is widely used and tested for improving the soil availability. However, mordenite is also known to improve soil available nutrients and water use efficiency (Stamatakis et al., 2017). Mordenite is a high silicate zeolite and large-pore zeolite with high surface area (Gosselink et al., 2010). In our study, mordenite application could improve the soil available N, P, and K; their uptake; dry matter yield; and WUE in Alfisols. Overall performance for both the years is shown in **Figure 11**. Uptake of nitrogen, phosphorus, and potassium by tomato is significantly affected by fertiliser levels and zeolite levels. An increase in fertiliser application leads to a decrease in nitrogen use efficiency and an increase in soil nitrate N accumulation and soil fertility. Combined supplementation of chemical fertilisers with zeolite had a notable effect on the chemical properties of the soil and also on yield. Zeolite prevents nutrients to be lost through leaching (Sun et al., 2019). Our study showed a strong correlation among N, P, and K availability in soil and their uptake in plant and dry matter yield and water use efficiency (**Figure 12**).

Application of zeolites in soil has gained importance for reducing N leaching and increase N efficiency. Sepaskhah and Yousefi (2007) reported that application of 2–8 g zeolite kg^−1^ prevented nutrient leaching in sandy loam soil. MesoLite zeolite has low surface area (9–12 m^2^ g^−1^) and very high cation exchange capacity [494 cmol(+)/kg]. The application of mesolite to sandy soil @ 0.4% resulted in reducing the NH_4_^+^ to the extent of 90% as compared to unamended soil (Zwingmann et al., 2009). In canola, the application of 270 kg N ha^−1^ without zeolite resulted in the greatest amount of N leaching loss to the tune of 144.23 kg ha^−1^ in sandy soils (Gholamhoseini et al., 2012). Due to its multiple positive effects on the chemical soil properties, zeolite contributes to improved nutrient, crop productivity, and crop quality. Water scarcity is a major limiting factor of agricultural production in arid locations mainly due to low precipitation and high evaporation and is aggravated by global climate change through extreme events and prolonged dry seasons. However, studies proved that zeolite improves water and plant nutrients. Recently, it is reported that the positive impact of zeolite application on paddy yield could continue even in the fifth year after its initial application. Moreover, application of zeolite @ 10 tonnes ha^−1^ (Z10) increased soil NH_4_^+^–N by 27%–38% and NO_3_–N by 14%–22% in 5 years compared to Z0 (control) (Zhao et al., 2023). Thus, zeolite application can last for 5 years. Thus, it can be used along with mineral fertilisers to earn more profits.

## 4 Conclusion

Zeolite is a promising amendment in soil management. In this study, natural zeolite sample purchased from local market was characterized using XRD and SEM to known the type of zeolite and found that it is mordenite zeolite. The sorption study revealed that mordenite zeolite has shown good adsorption of ammonical-N and to lesser extent of P and K, which was also found to have a good fit in Langmuir and Freundlich isotherms with high R^2^. This suggests that mordenite has good affinity for ammonical-N and also, in a little extent, for K. Leaching column study also revealed that it was effective in mitigating the losses under laboratory condition. Mainly, combined application of zeolite and N supplemented through urea could mitigate N leaching loss and ensure slow release availability when applied along with urea. There was a significant impact on soil available nutrients (N, P, and K) due to application of 100% RDF along with zeolite @ 200 kg ha^−1^ with mean increase of 46.9%, 63.6%, and 40.6%, respectively, under 100% FC, while the mean increase in soil available N, P, and K was up to 39.5%, 68%, and 21%, respectively, under 50% FC over 100% RDF without zeolite. There was also a significant positive impact on plant nutrients (N, P, and K) uptake, dry matter production, and water use efficiency due to application of 100% RDF along with zeolite @ 200 kg ha^−1^ under 50% and 100% FC as compared to without zeolite application. Thus, mordenite zeolite can also be an option to improve soil’s water-holding capacity and nutrient use efficiency by reducing the leaching losses and improving the crop yield. However, comprehensive studies across different agro climatic conditions and its beneficial effect on soil quality need to explore.

## Data Availability

The original contributions presented in the study are included article/supplementary material. Further inquiries can be directed to the corresponding author.
